# The replication-competent HIV reservoir is a genetically restricted, younger subset of the overall pool of HIV proviruses persisting during therapy, which is highly genetically stable over time

**DOI:** 10.21203/rs.3.rs-3259040/v1

**Published:** 2023-08-16

**Authors:** Aniqa Shahid, Signe MacLennan, Bradley R. Jones, Hanwei Sudderuddin, Zhong Dang, Kyle Cobamibias, Maggie C. Duncan, Natalie N. Kinloch, Michael J. Dapp, Nande M Archin, Margaret A. Fischl, Igho Ofotokun, Adaora Adimora, Stephen Gange, Bradley Aouizerat, Mark H. Kuniholm, Seble Kassaye, James I. Mullins, Harris Goldstein, Jeffrey B. Joy, Kathryn Anastos, Zabrina L. Brumme

**Affiliations:** Faculty of Health Sciences, Simon Fraser University, Burnaby, BC, Canada; Faculty of Health Sciences, Simon Fraser University, Burnaby, BC, Canada; British Columbia Centre for Excellence in HIV/AIDS, Vancouver, BC, Canada; British Columbia Centre for Excellence in HIV/AIDS, Vancouver, BC, Canada; British Columbia Centre for Excellence in HIV/AIDS, Vancouver, BC, Canada; British Columbia Centre for Excellence in HIV/AIDS, Vancouver, BC, Canada; Faculty of Health Sciences, Simon Fraser University, Burnaby, BC, Canada; Faculty of Health Sciences, Simon Fraser University, Burnaby, BC, Canada; Department of Microbiology, University of Washington, School of Medicine, Seattle, WA, USA; UNC HIV Cure Center, Institute of Global Health and Infectious Diseases, University of North Carolina at Chapel Hill, NC, USA; Department of Medicine, University of Miami School of Medicine, Miami, FL, USA; Division of Infectious Diseases, Department of Medicine, Emory University School of Medicine, Atlanta, GA, USA; Departments of Medicine and Epidemiology, University of North Carolina School of Medicine, UNC Gillings School of Global Public Health, Chapel Hill, NC, USA; Department of Epidemiology, Johns Hopkins Bloomberg School of Public Health, Baltimore, MD, USA; College of Dentistry, New York University, New York, NY, USA; Department of Epidemiology and Biostatistics, University at Albany, State University of New York, Rensselaer, New York, NY, USA; Division of Infectious Diseases and Tropical Medicine, Georgetown University, Washington, DC, USA; Department of Microbiology, University of Washington, School of Medicine, Seattle, WA, USA; Departments of Microbiology and Immunology and Pediatrics, Albert Einstein College of Medicine, Bronx, New York, NY, USA; British Columbia Centre for Excellence in HIV/AIDS, Vancouver, BC, Canada; Department of Medicine, Albert Einstein College of Medicine, New York, NY, USA; Faculty of Health Sciences, Simon Fraser University, Burnaby, BC, Canada

**Keywords:** HIV, reservoir, persistence, phylogenetics, rebound

## Abstract

Within-host HIV populations continually diversify during untreated infection, and members of these diverse forms persist within infected cell reservoirs, even during antiretroviral therapy (ART). Characterizing the diverse viral sequences that persist during ART is critical to HIV cure efforts, but our knowledge of on-ART proviral evolutionary dynamics remains incomplete, as does our understanding of the differences between the overall pool of persisting proviral DNA (which is largely genetically defective) and the subset of intact HIV sequences capable of reactivating. Here, we reconstructed within-host HIV evolutionary histories in blood from seven participants of the Women’s Interagency HIV Study (WIHS) who experienced HIV seroconversion. We measured diversity, lineage origins and ages of proviral sequences (*env-gp120*) sampled up to four times, up to 12 years on ART. We used the same techniques to study HIV sequences emerging from the reservoir in two participants. Proviral clonality generally increased over time on ART, with clones frequently persisting across multiple time points. The integration dates of proviruses persisting on ART generally spanned the duration of untreated infection (though were often skewed towards years immediately pre-ART), while in contrast, reservoir-origin viremia emerging in plasma was exclusively “younger” (i.e., dated to the years immediately pre-ART). The genetic and age distributions of distinct proviral sequences remained highly stable during ART in all but one participant in whom, after 12 years, there was evidence that “younger” proviruses had been preferentially eliminated. Analysis of within-host recombinant proviral sequences also suggested that HIV reservoirs can be superinfected with virus reactivated from an older era, yielding infectious viral progeny with mosaic genomes of sequences with different ages. Overall, results underscore the remarkable genetic stability of distinct proviral sequences that persist on ART, yet suggest that replication-competent HIV reservoir represents a genetically-restricted and overall “younger” subset of the overall persisting proviral pool in blood.

## Author Summary

Characterizing the genetically-diverse HIV sequences that persist in the reservoir despite antiretroviral therapy (ART) is critical to cure efforts. The ages and lineage origins of these sequences can be elucidated if they are interpreted in context of HIV’s within-host evolutionary history, which can help us understand reservoir composition and persistence. Our results confirm that proviruses persisting on ART, which are largely genetically defective, broadly reflect the extent of within-host HIV evolution pre-ART, and that the clonal expansion that commonly occurs during ART is not appreciably accompanied by the loss of distinct proviral lineages. Rather, on-ART proviral genetic composition remained remarkably stable in all but one participant, in whom there was evidence that, by the twelfth year of ART, some proviruses that had integrated near ART initiation had been preferentially eliminated from blood. Analysis of within-host recombinants supports the notion that reservoir cells can become superinfected with HIV reactivated from older infection eras, yielding mosaics of sequences of different ages. Our findings also strongly suggest that the replication-competent reservoir in blood is a genetically restricted, “younger” subset of all persisting proviruses. If so, cure strategies will need to eliminate a reservoir that differs in key respects from the overall proviral pool.

## Introduction

The ability of HIV to persist as an integrated provirus within a small fraction of infected cells, even during suppressive antiretroviral therapy (ART), is the main barrier to cure [1, 2]. It is also the reason why ART must be taken for life. Seeding of HIV sequences into these reservoir cells begins immediately following infection [3, 4] and continues until viral suppression is achieved on ART, thereby establishing a genetically diverse viral reservoir [5–9]. Understanding the within-host evolutionary dynamics of the proviruses that persist during ART, as well as the origins of HIV sequences that emerge from the reservoir if ART is interrupted, will aid the development of curative strategies.

In recent years, our understanding of reservoir dynamics has been enriched by studies that have interpreted on-ART proviral genetic diversity in blood in the context of HIV’s within-host evolutionary history [5–7, 10–12]. These studies have revealed that a large percentage of proviruses that persist in the blood during ART (most of which are genetically defective [13–15]), as well as the vast majority of replication-competent reservoir sequences that persist during this time, “date” to the year or two preceding ART initiation [5, 6, 9–11]. We now understand that this is because reservoir turnover during untreated infection is relatively rapid (the half-life of persisting proviruses during this period is estimated to be a year or less [5, 16]), which means that, if ART is not initiated until advanced chronic infection, many of the earliest within-host lineages will have already been eliminated by this time. Nevertheless, proviruses dating to earlier periods of infection are routinely recovered during ART, albeit less frequently [5–7, 9, 11].

During the initial years of ART, the proviral pool slowly decreases in size (initial on-ART half-lives of intact and defective proviruses are ~ 4 and > 10 years respectively, with decay slowing further thereafter [17–19]). At the same time, clonal expansion of infected cells also occurs [20–22]. Given these opposing processes, and assuming that no new viral variants are seeded into the reservoir during ART [5, 23], it is reasonable to hypothesize that the persisting proviral pool will gradually decline in genetic diversity, as distinct proviruses are eliminated over time. Relatively few studies, however, have investigated on-ART proviral genetic stability [20, 24–29], of which only two have done so in the context of HIV’s within-host evolutionary history [5, 6]. Doing so can shed light on the lineage origins and ages of persisting proviruses, but the results of these two studies were not entirely concordant: while one suggested that “younger” HIV lineages may be preferentially eliminated during the initial years of ART (though this did not reach statistical significance [6]), the other supported relative proviral genetic stability even in the longer-term (though the primary goal of the latter analysis was to investigate whether residual HIV replication occurs during ART, not to evaluate proviral genetic stability over time [5]). Even fewer studies have compared the within-host evolutionary origins and ages of proviruses persisting on ART with those of HIV sequences emerging from the reservoir (i.e., as rebound viremia) [30], which have been shown to include within-host recombinants of unknown origin [31]. Such analyses can help illuminate how the rebound-competent reservoir in blood may be distinctive from the overall, largely defective, proviral pool.

To address these knowledge gaps, we reconstructed within-host HIV evolutionary histories in seven participants enrolled in the Women’s Interagency HIV Study (WIHS) who seroconverted during follow-up in order to investigate the genetic stability of distinct proviruses sampled up to four times, up to 12 years following ART initiation. In two participants, we also investigated the diversity and age distribution of reservoir-origin HIV sequences that emerged in plasma post-ART.

## Results

### Participant characteristics and sampling

**Figures 1:**
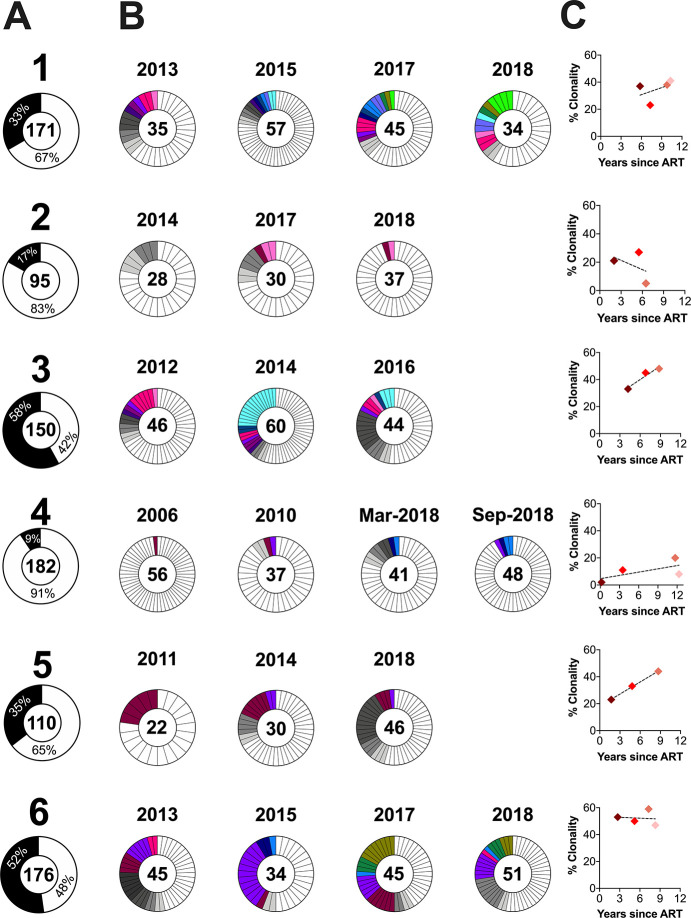
Participant sampling timeline. Time zero denotes ART initiation. The right arrow denotes enrolment into cohort, while the asterisk denotes the clinically estimated date of infection, defined as the midpoint between the last negative and first positive HIV tests. Grey shading denotes ART. Here and throughout all figures, closed circles denote pre-ART plasma HIV RNA sampling. Open circles denote post-ART plasma HIV RNA sampling. Diamonds denote proviralsampling on ART. Colors of symbols match those used in Fig 3, Fig 5, Fig 6, Fig 7, Fig 8, Fig 9, S5 Fig, S8 Fig and S9 Fig. Participants 1, 3 and 7 correspond to participants 1, 3 and 6 in Dapp *et al* [32].

We reconstructed the within-host HIV evolutionary histories of seven women who initiated ART a median 9 (range 1.9–12) years following their estimated infection dates, which were calculated as the midpoint between the last negative and first seropositive HIV visits (Fig. 1; Table 1). We then leveraged this information to characterize the genetics and dynamics of proviruses and reservoir-origin plasma HIV sequences sampled longitudinally during ART. Together, we analyzed 1,092 single-genome-amplified intact HIV RNA *env-gp120* sequences (median 181, range 50–239 per participant) from a median 9 (range 2–13) plasma samples collected over a median 8.3 (range 0.8–11.8) years pre-ART (where these sequences were previously published for three participants [32]), along with 926 intact proviral *env-gp120* sequences (median 150, range 42–182 per participant) from a median 3 time points (range 1 –4) spanning a median 8.7 (range 2.8–12.3) years during ART (Fig. 1). For participant 1 we also analyzed 114 plasma HIV RNA *env-gp120* sequences isolated during an ART interruption, and from participant 5 we also analyzed four plasma HIV RNA *env-gp120* sequences isolated during initial loss of viral control. All participants had HIV subtype B, with no evidence of dual or superinfection (S1 Fig). As expected [8, 32–34], the overall extent of within-host HIV diversity correlated strongly with the duration of untreated infection (Spearman’s ρ = 0.85, p = 0.03;S1 Fig, *inset).*

### Proviral clonal dynamics during the initial years of ART

**Figures 2:**
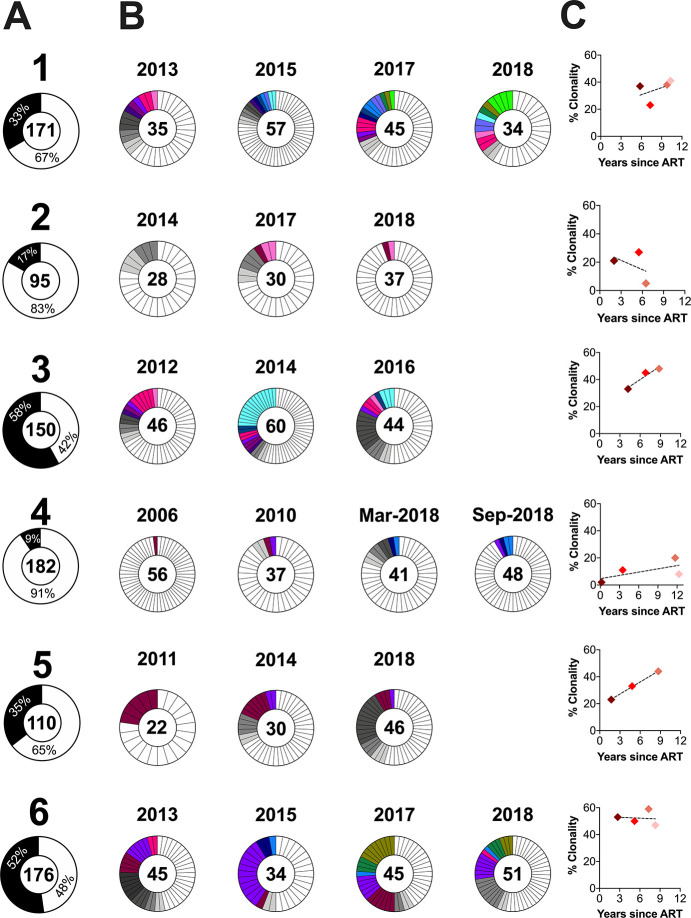
Participant sampling timeline. Time zero denotes ART initiation. The right arrow denotes enrolment into cohort, while the asterisk denotes the clinically estimated date of infection, defined as the midpoint between the last negative and first positive HIV tests. Grey shading denotes ART. Here and throughout all figures, closed circles denote pre-ART plasma HIV RNA sampling. Open circles denote post-ART plasma HIV RNA sampling. Diamonds denote proviralsampling on ART. Colors of symbols match those used in Fig 3, Fig 5, Fig 6, Fig 7, Fig 8, Fig 9, S5 Fig, S8 Fig and S9 Fig. Participants 1, 3 and 7 correspond to participants 1, 3 and 6 in Dapp *et al* [32].

For participants 1 through 6, we sampled proviruses at a minimum of three time points during the initial years of ART, allowing us to investigate clonal dynamics. The overall percentage of putatively clonal sequences, defined as those that matched at least one other sequence with 100% nucleotide identity in *env-gp120,* ranged from 9% (participant 4) to 58% (participant 3) (Fig. 2A). In all participants, we recovered clones that were observed at only one on-ART time point (grey slices, Fig. 2B) and across more than one time point (colored slices, Fig. 2B). In participants 3, 5 and 6, we recovered at least one clone that persisted across all on-ART time points. The proportion of clonal proviruses increased or remained stable over time in five of six participants (Fig. 2C).

### Proviral ages, within-host origins and dynamics on ART

We next used a phylogenetic approach [7] to investigate the ages, within-host evolutionary origins and dynamics of proviral lineages persisting on ART. Only intact, non-hypermutated sequences that showed no evidence of within-host recombination were included in this analysis (recombinants were analyzed separately; see below). To mitigate the inherent uncertainty in within-host HIV evolutionary reconstruction, and to allow us to estimate error in the parameters of interest (e.g., proviral integration dates; population structure), we inferred a minimum 1,500 trees per participant, and conditioned results across all trees. We rooted each tree at the location that maximized the correlation between the root-to-tip distances of the pre-ART plasma HIV RNA sequences and their sampling dates (as within-host sequence divergence from the transmitted/founder virus increases over time during untreated infection [32, 34, 35]). This root location represents the most recent common ancestor of the dataset, which should be the transmitted/founder virus (or a close descendant) in this cohort of seroconverters. We then fit a linear model to each tree relating the root-to-tip genetic distances of distinct pre-ART plasma *env-gp120* sequences to their sampling dates. Here, the slope represents the within-host pre-ART *env-gp120* evolutionary rate, and the x-intercept represents the phylogenetically-estimated infection date. This linear model was then used to convert the root-to-tip distance of each post-ART sequence of interest to its integration date. Each sequence’s estimated integration date was then averaged over all within-host trees that passed quality control (QC) (see methods), and reported along with its 95% highest posterior density (HPD) interval.

### Participant 1

**Figures 3:**
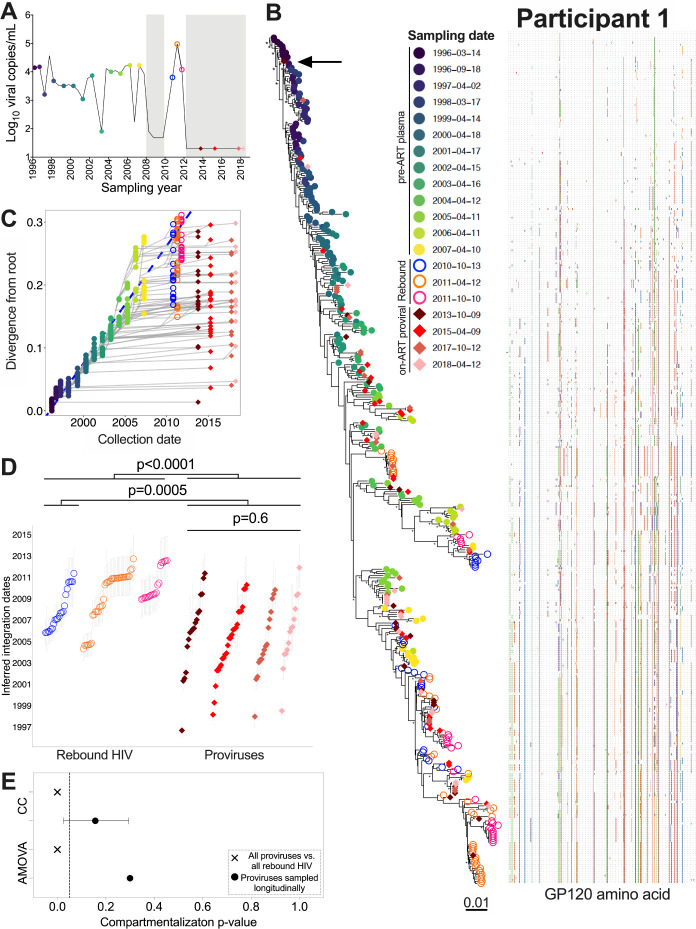
Participant 1: diversity and inferred integration dates of HIV sequences persisting during ART. (A) Plasma viral load history, with symbols denoting sampling time points. For viral load assays performed during 2008 to 2010, the lower limit of quantification (LLOQ) was either 80 or 48 HIV RNA copies/mL, and thereafter it was 20 copies HIV RNA copies/mL. Closed circles denote pre-ART plasma HIV RNA sampling, open circles denote post-ART plasma HIV RNA sampling and diamonds denote proviral sampling on ART Grey shading denotes ART (B) Example rooted within-host phylogeny, with scale in estimated substitutions per nucleotide site. Asterisks identify nodes supported by posterior probabilities ≥70%. The adjacent highlighter plot shows ENV-GP120 amino acid diversity, with colored ticks denoting non-synonymous substitutions with respect to the reference sequence at the top of the phylogeny. (C) Linear model (dashed blue diagonal) relating plasma HIV RNA collection dates (closed colored circles) to their respective root-to-tip distances in the example phylogeny shown in (B). This linear model is then used to convert the root-to-tip distances of on-ART sequences of interest (rebound HIV in open circles and proviruses in colored diamonds) to their integration dates. Grey lines trace the phylogenetic relationships between HIV *env-gp120* sequences. (D) Integration dates and 95% HPD intervals for distinct on-ART sequences, stratified by collection year, that were derived from averaging results across all QC-passed phylogenies for this participant. Comparison of integration dates across all the longitudinal proviral on-ART time points is compared using a Kruskal-Wallis test, while comparisons of plasma versus proviral integration dates are computed using the Mann-Whitney U-test. (E) p-values from the tests for population structure (AMOVA = Analysis of Molecular Variance; CC = Correlation Coefficient) comparing distinct proviral sequences per time point (closed black circle) or between all proviruses to all rebound viruses (cross symbol). For CC, the bars represent the 95% HPD interval of the p-values derived from all QC-passed phylogenies for this participant.

Participant 1 was estimated to have acquired HIV in late 1995, but only initiated ART in Jan 2008 (Fig. 1, Fig. 3A). We inferred 15,000 within-host phylogenies relating 207 plasma HIV RNA *env-gp120* sequences collected over 12 years of untreated infection (these sequences were originally published in [32]), 97 plasma HIV RNA *env-gp120* sequences collected at three time points during an initial ART interruption, and 90 proviral *env-gp120* sequences collected at four subsequent time points after viremia was re-suppressed on ART (Table 1; S1 Table). Of these trees, 7,218 passed quality control (QC) (see methods and S1 Table). The trees exhibited the ladder-like shape typical of within-host HIV evolution, where plasma HIV sequences sampled during untreated infection were increasingly divergent from the root over time (example phylogeny in Fig. 3B). This shape is the result of serial genetic bottlenecks imposed by immune responses from which the virus continually escapes; the selective sweeps that characterize this process can be seen in the adjacent amino acid highlighter plot. The linear model relating the root-to-tip distances of pre-ART plasma HIV RNA sequences in this tree to their collection dates is shown in Fig. 3C, where the slope of this line represents the within-host pre-ART *env-gp120* evolutionary rate and the x-intercept represents the phylogenetically-estimated infection date. When we conditioned these metrics over all QC-passed trees, the mean root date was December 1995 (95% HPD, August 1995 to March 1996), consistent with the participant’s clinically-estimated infection date, and the mean *env-gp120* evolutionary rate was 4.8×10^−5^ (95% HPD 3.8×10^−5^ – 6.0×10^−5^) substitutions per nucleotide site per day (S1 Table). Note that all subsequent analyses are restricted to distinct HIV sequences per time point, as our overarching goal was to examine the diversity and ages of distinct proviral sequences in a way that it not influenced by clonal expansion.

Participant 1 interrupted ART following initial suppression. This allowed us to investigate the within-host evolutionary origins of the HIV sequences that rebounded in plasma, which we sampled at three time points between October 2010 and October 2011. All sequences sampled at the initial time point interspersed with plasma sequences that circulated in the three years prior to ART (Fig. 3B), and “dated” to between late 2005 and the time of sampling (Fig. 3C, Fig. 3D). Sequences subsequently sampled in April and October 2011 also dated to this era, which is expected given that at least some of these are descendants of the initial rebounding population (S2 Fig shows an enlarged portion of the phylogeny, where likely descendants are indicated by green arrows, including the large clade at the bottom). Nevertheless, a clade of closely-related rebound sequences isolated in April 2011 was less divergent from the root and distinct from those sampled the previous October (S2 Fig, black bracket), an observation that was consistent across all trees (see consistently earlier integration dates for these sequences in Fig. 3D). This suggests that these were not descendants of the initial rebounding population, but rather descendants of a provirus or clonal infected cell population that reactivated independently near this time.

In striking contrast to the rebound HIV sequences, participant 1’s distinct proviral sequences subsequently sampled in 2013, 2015, 2017 and 2018 interspersed throughout the entire phylogeny, nearly all the way back to the root (Fig. 3B). Though the inferred integration dates of these proviruses were slightly skewed towards the years leading up to ART, as well as the subsequent ART interruption period, numerous older proviruses were recovered, including one dating to September 1996, less than a year following infection (Fig. 3D). The integration date distributions of distinct proviruses sampled longitudinally on ART were highly stable over time (Kruskal-Wallis p = 0.6; Fig. 3D). Notably, these proviruses were overall significantly older than those that had previously rebounded in plasma (Mann Whitney p = 0.0005 when comparing the ages of all proviruses vs. those of the initially rebounding population; p < 0.0001 when comparing all proviruses to the entire rebounding population). We also observed proviruses that were identical or near-identical to sequences that had previously rebounded in plasma (see S2 Fig, black arrows), consistent with reservoir re-seeding during this rebound event.

We next investigated whether the distinct proviral sequences sampled during ART showed any evidence of changing population structure over time. This could happen, for example, if distinct within-host lineages were being eliminated during the initial years of ART, such that later-sampled populations represented a genetically restricted subset of earlier-sampled ones, or if proviral populations sampled at different time points were distinct from one another (which could occur, for example, if reservoir cells re-entered blood from a compartmentalized tissue population). Neither test revealed significant evidence of population structure: Analysis of Molecular Variance (AMOVA) yielded p = 0.3, while the Correlation Coefficient (CC) test, conditioned over all passing trees, yielded a mean p = 0.16 (Fig. 3E). Therefore, even though proviral clonality increased during the first 9 years of ART (Fig. 2C), the number and composition of distinct proviral lineages remained consistent over time. In contrast, plasma rebound HIV sequences were significantly compartmentalized compared to the overall proviral pool (p = 0 for both AMOVA and CC), indicating that only a restricted subset of persisting proviruses re-seeded viremia at that time.

Within-host recombinants, which are an important source of within-host HIV diversity [36, 37], cannot be dated using the phylogenetic approaches used here. For recombinants we instead used RDP4 [38] to obtain the origin date of each parental sequence component.

We were particularly interested in the possibility of recombination between sequences from different infection eras, a phenomenon that has been predicted by mathematical modeling [39] as this would indicate that reservoir cells can be superinfected with HIV reactivated from an older era.

**Figure 4:**
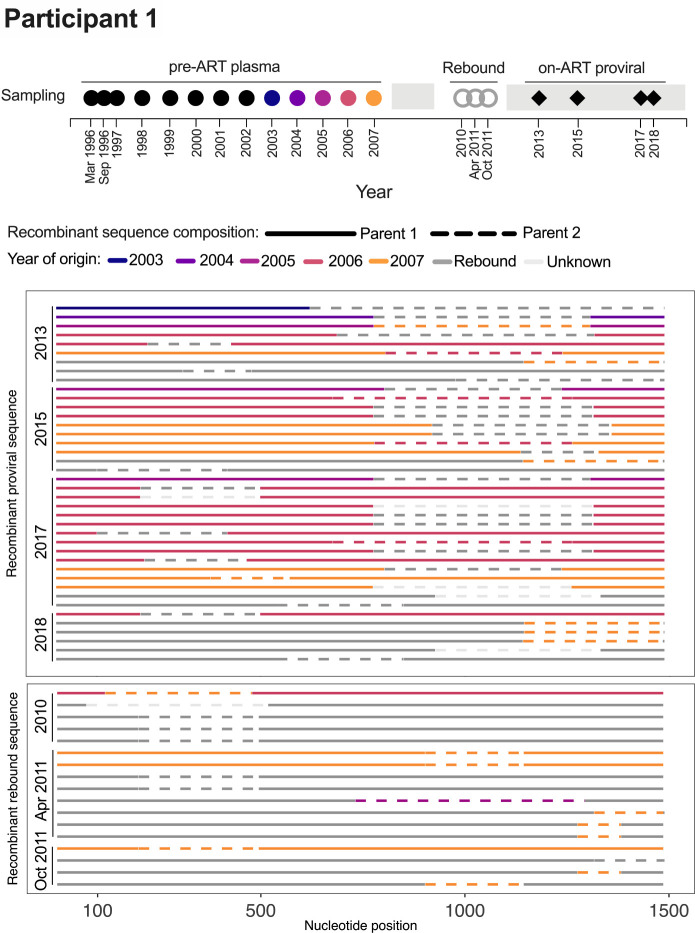
Participant 1: recombinant *env-gp120* proviral and rebound sequences. Colored circles in the sampling timeline (top) denote the year of origin of one or more recombinant sequences (below). Recombinant sequences are grouped by type and year of collection, with solid and dotted lines representing the two parent sequences, colored by year of origin.

In participant 1, we identified 40 distinct recombinant *env-gp* 720 proviral sequences that were sampled on ART between 2013–2018 (Fig. 4). Of these, none contained a parent sequence from the first 7 years of infection. Rather, 5 were mosaics of sequences that rebounded during the 2010–2011 treatment interruption, 6 were mosaics of sequences that circulated in the three years prior to ART, 22 were mosaics of rebound sequences and pre-ART sequences from 2005–2007, which fueled the rebound event, and 5 were sequences whose minor parent could not be identified. Two mosaics comprising 2003 and rebound sequences were observed, but these parent sequences could plausibly have circulated at the same time, as the root-to-tip divergences of sequences from these periods overlapped one another (Fig. 3C). As such, there was no convincing evidence in this participant of recombinants between viruses from substantially different infection eras. Similarly, the 17 recombinants identified among the 2010–2011 plasma rebound sequences were all mosaics of rebound sequences and/or pre-ART sequences that fueled the rebound (Fig. 4).

### Participant 2

**Figure 5:**
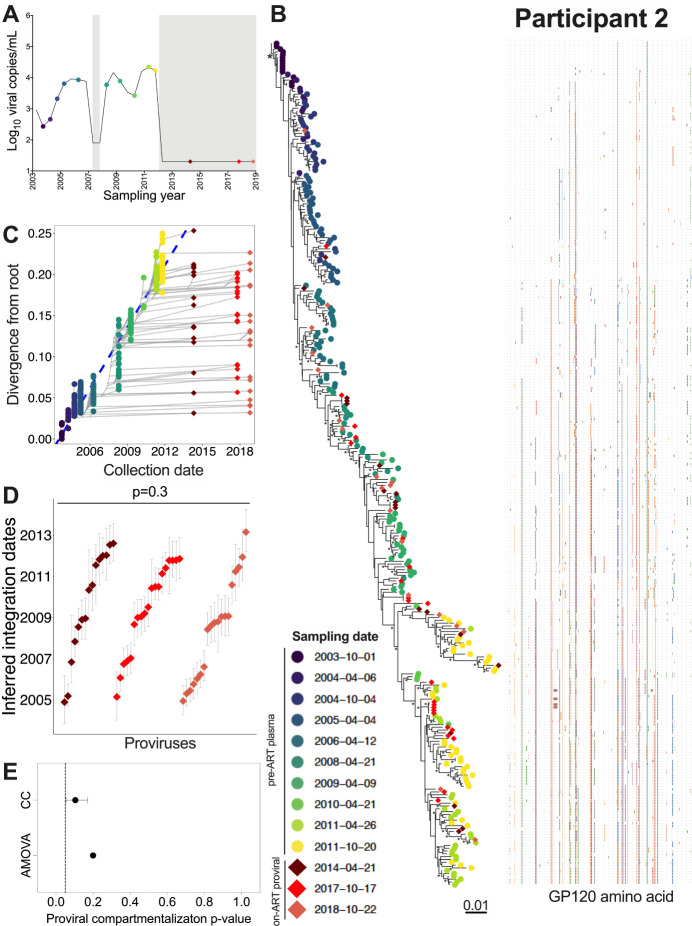
Participant 2: diversity and inferred integration dates of HIV sequences persisting during ART. Legend as in Fig 3, except that (E) shows p-values from genetic compartmentalization tests applied to serially-sampled proviruses only.

Participant 2 was estimated to have acquired HIV in January 2003 (Table 1). Though ART was briefly initiated in 2007, durable suppression was not achieved until ART was re-initiated in January 2012 (Table 1; Fig. 5A). We inferred 5,250 phylogenies from 239 plasma HIV RNA *env-gp120* sequences isolated during untreated infection, along with 61 proviral *env-gp120* sequences sampled at 2-, 6-, and 7-years post-ART. Of these, 3,842 trees passed QC, yielding a mean root date of May 2003 (95% HPD, January to September 2003; S1 Table). Proviral sequences sampled during ART interspersed throughout nearly the whole tree (example phylogeny and root-to-tip divergence plot in Fig. 5B, Fig. 5C), where the oldest provirus dated to late 2004, approximately two years after infection (Fig. 5D). The integration date distributions of distinct proviruses sampled over seven years on ART remained consistent over time (Kruskal-Wallis p = 0.3; Fig. 5D) and showed no evidence of temporal population structure (AMOVA p = 0.2, CC mean p = 0.1; Fig. 5E).

Four recombinant proviral sequences were recovered from participant 2, three of which were mosaics of sequences that circulated in the two years prior to ART (S3A Fig). Of note, one recombinant had a 5’ half that dated to 2006 and a 3’ half that dated to 2010 (S3A Fig; asterisk), years where the root-to-tip divergence measurements of sampled sequences did not overlap (Fig. 5C). The recovery of this sequence suggests that reservoir cells can be superinfected with HIV from a different infection era, yielding infectious progeny with mosaic genomes of sequences with different ages.

### Participant 3

**Figure 6:**
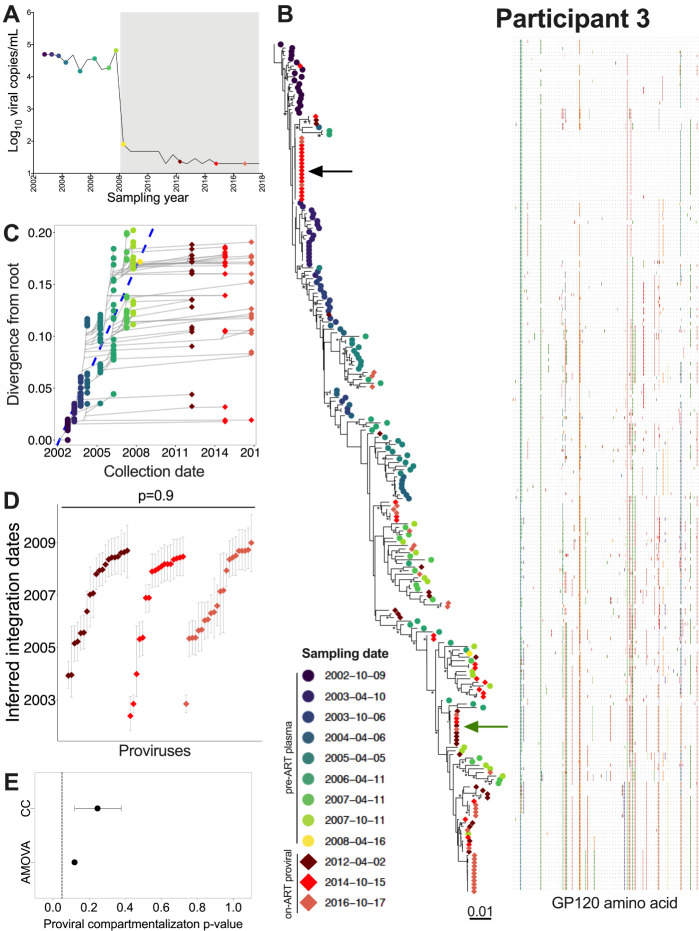
Participant 3: diversity and inferred integration dates of HIV sequences persisting during ART. Legend as in Fig 5.

Participant 3 was estimated to have acquired HIV in July 2002 and initiated ART in January 2008 (Table 1; Fig. 6A). We inferred 4,500 phylogenies from 140 pre-ART plasma *env-gp120* sequences (published in [32]) along with 96 proviral sequences sampled after 4, 7, and 9 years on ART. All passed QC, yielding a mean root date of November 2001 (95% HPD, July 2001 to March 2002), which was slightly earlier than the clinically-estimated infection date of July 2002 (S1 Table). Again, distinct proviral sequences sampled on ART interspersed throughout the tree (example phylogeny and divergence plot shown in Fig. 6B, Fig. 6C). We observed a number of expanded clones, including one relatively near the root that we recovered at both later on-ART time points (Fig. 6B, black arrow) and another more divergent one that we recovered at all on-ART time points (Fig. 6B, green arrow). The oldest recovered provirus, isolated in 2014, was estimated to have integrated in May 2002 (Fig. 6D). The integration date distributions of distinct proviruses were stable during ART (Kruskal-Wallis p = 0.9; Fig. 6D), with no evidence of changing population structure (AMOVA p = 0.1; CC mean p = 0.25; Fig. 6E). All 12 recombinant proviral sequences collected during ART were mosaics of sequences that circulated in the three years prior to ART (S3B Fig).

### Participant 4

**Figure 7:**
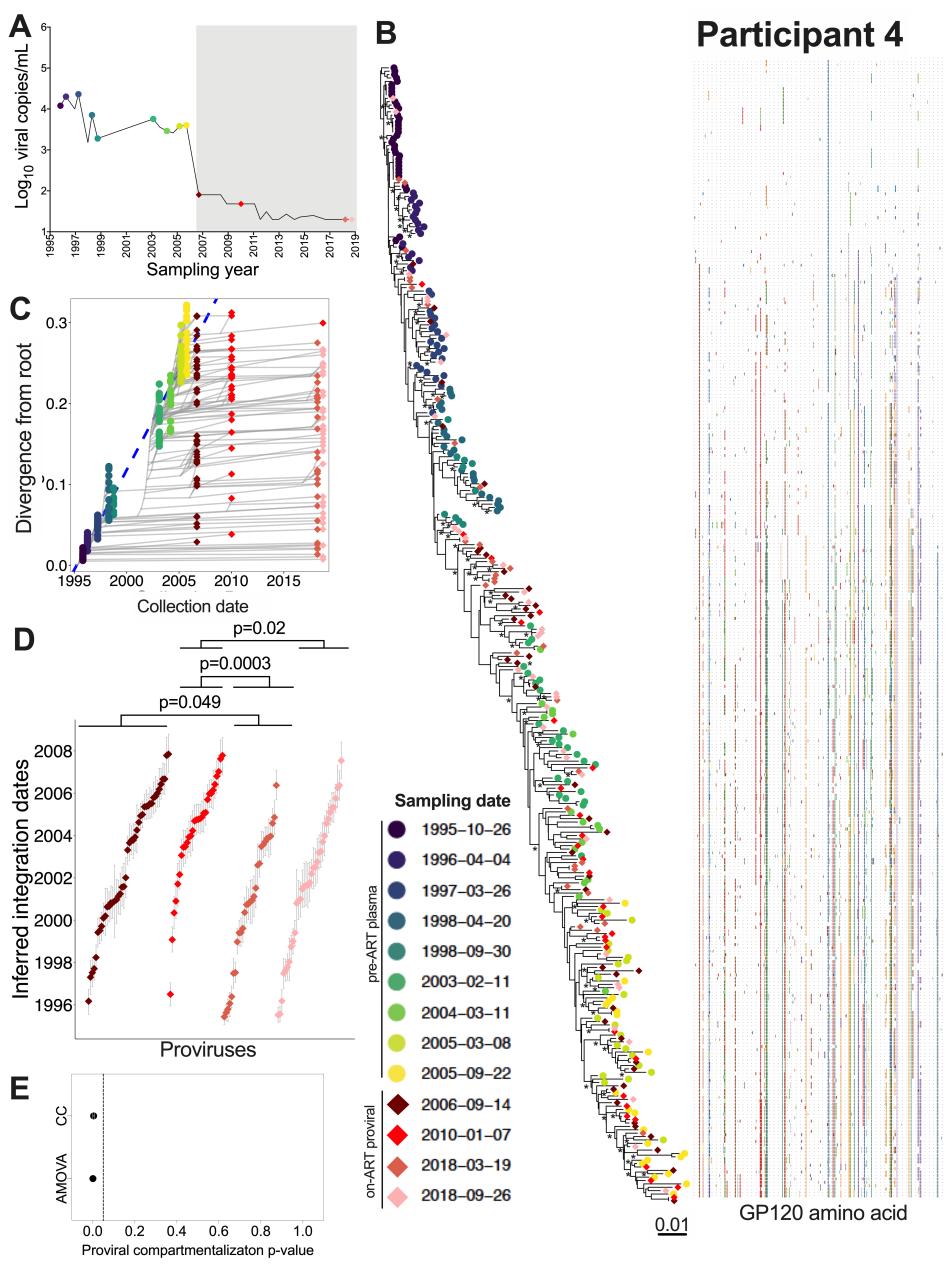
Participant 4: diversity and inferred integration dates of HIV sequences persisting during ART. Legend as in Fig 5, except that (D) shows pairwise comparisons between groups after correction for multiple comparisons (as the overall Kruskal-Wallis test returned p=0.0004).

Participant 4 was estimated to have acquired HIV in July 1995 and initiated suppressive ART in June 2006 (Table 1; Fig. 7A). We inferred 6,000 within-host phylogenies from 195 pre-ART plasma *env-gp120* sequences along with 141 proviral *env-gp120* sequences sampled at four time points up to 12 years post-ART (Table 1). All trees passed QC (example phylogeny and divergence plot in Fig. 7B and Fig. 7C), yielding a mean estimated root date of February 1995 (95% HPD, October 1994 to June 1995; S1 Table), which was only slightly earlier than the clinically-estimated infection date. Proviruses isolated during ART had integrated throughout the entire course of untreated infection and included 5 distinct sequences, isolated in March and September 2018, that dated to 1995 (i.e., the first six months after transmission). Of note, and in contrast to participants 1 –3, the age distributions of distinct proviruses sampled in March and September 2018 (~ 12 years after ART initiation, the longest follow-up of any participant in the study) were on average older than those sampled in the earlier years of ART (Kruskal-Wallis p = 0.0004; pairwise post-test p-values shown in Fig. 7D). Both AMOVA (p = 0.001) and CC (mean p = 0.004) also supported a change in proviral population structure over this period (Fig. 7E).

Eleven recombinant proviral sequences were recovered from participant 4, ten of which were mosaics of sequences that circulated in the three years prior to ART initiation (S4A Fig). Of note, and similar to participant 2, one recombinant was a mosaic of sequences that were inferred to have circulated in the years 2000 and 2005 (S4A Fig; asterisk), years where the root-to-tip divergence measurements of sampled sequences would not have overlapped (Fig. 7C). This again suggests that reservoir cells can be superinfected with HIV from a different infection era.

### Participant 5

**Figure 8:**
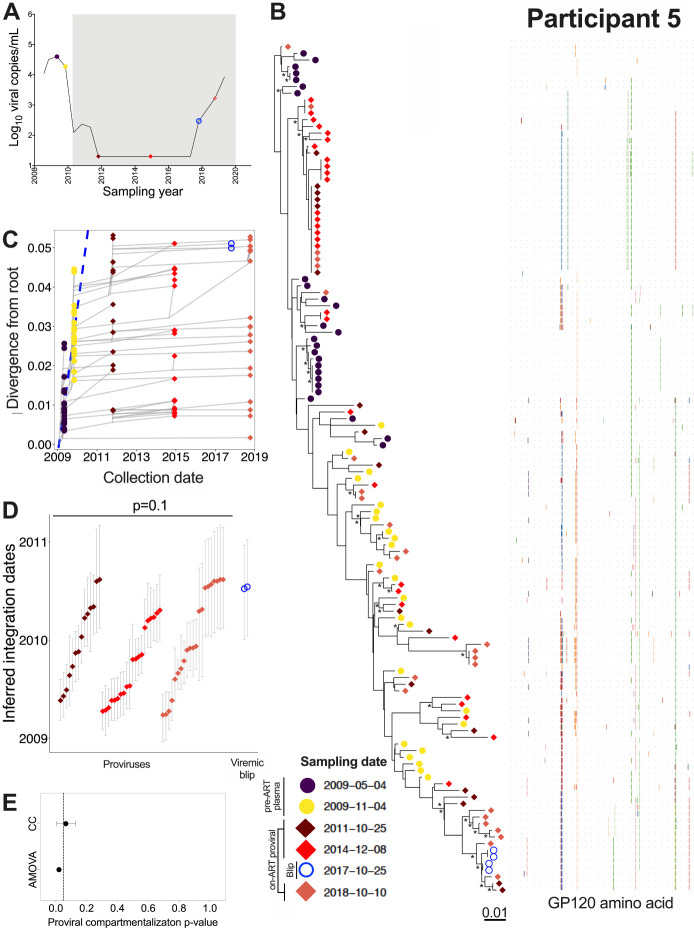
Participant 5: diversity and inferred integration dates of HIV sequences persisting during ART. Legend as in Fig 5.

Participant 5 acquired HIV around March 2008 and initiated ART in February 2010 (Table 1; Fig. 8A). We inferred 1,500 phylogenies relating 50 *env-gp120* sequences collected at two pre-ART time points, 48 proviral *env-gp120* sequences collected at 2 and 5 years after ART initiation, 4 HIV RNA *env-gp120* sequences from an initial viremia increase to 295 HIV RNA copies/mL in October 2017, during the seventh year of ART, and 26 proviral sequences collected in year 9, after viral control was lost (Table 1). All phylogenies passed QC (S1 Table) and yielded a mean estimated root date of December 2008 (95% HPD, October 2008 to February 2009), suggesting that we did not reconstruct all the way back to transmission (example phylogeny and divergence plot in Fig. 8B, Fig. 8C). Similar to participant 1, phylogenetic dating revealed that proviruses sampled during ART integrated throughout untreated infection (with the earliest dating to May 2009, about a year after transmission), yet plasma HIV RNA sequences isolated during the initial loss of viremia control in year 7 of ART were located within the most distant clade from the root, and dated to around ART initiation (Fig. 8B). Although the age difference between the emerging plasma viruses and the overall proviral pool did not reach statistical significance (as only two of the four emerging HIV sequences were genetically distinct; Mann-Whitney p = 0.11), the emerging plasma viruses were clearly younger than the overall proviral pool (Fig. 8D). Comparison of the integration date distributions of proviruses sampled after 2, 5 and 9 years of ART revealed that these were not significantly different from one another (Kruskal-Wallis p = 0.1), though the year 9 proviral pool featured a slightly larger number of “younger” proviruses than the first two time points (Fig. 8D). The p-values of the population structure tests suggested a similar shift (AMOVA yielded p = 0.02 and CC yielded a mean p = 0.06; Fig. 8E), though these observations did not reach our pre-defined significance requirement of p < 0.05 on both tests. Overall, these results indicated that participant 5’s proviral pool was largely stable in terms of genetic composition, though the recently-emerging, “younger” HIV sequences in plasma may have re-seeded the reservoir to a modest extent. No recombinant sequences were recovered from this participant.

### Participant 6

**Figure 9:**
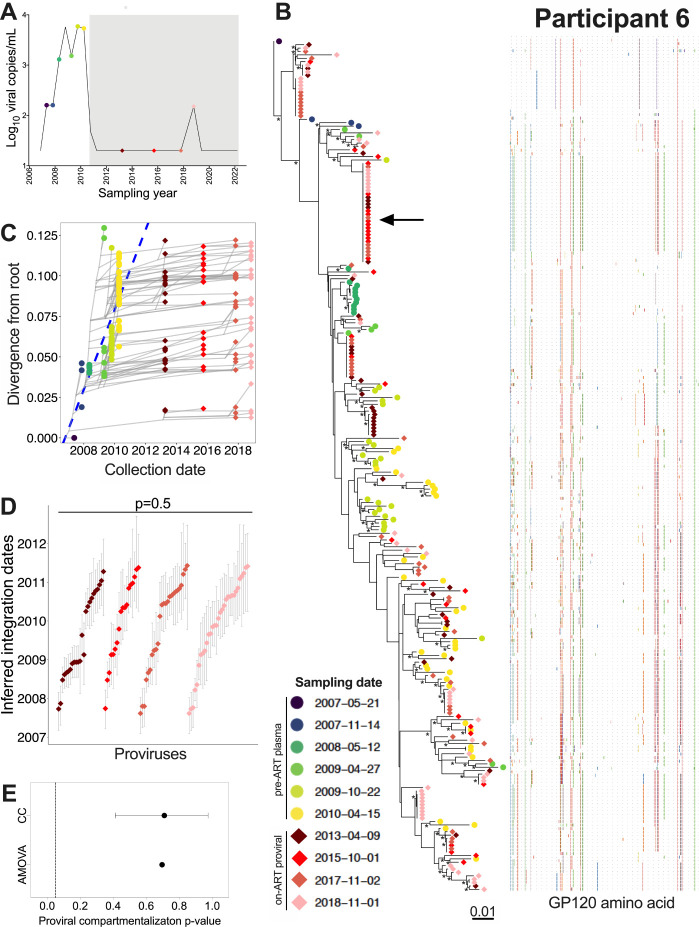
Participant 6: diversity and inferred integration dates of HIV sequences persisting during ART. Legend as in Fig 5.

Participant 6 acquired HIV around August 2006 and initiated ART in July 2010 (Table 1, Fig. 9A). We inferred 3,000 phylogenies relating 80 plasma *env-gp120* sequences isolated at 6 time points during untreated infection, and 161 proviral *env-gp120* sequences collected at 3-, 5-, 7- and 8-years post-ART. Of these, 2,528 passed QC and yielded a mean root date of January 2007 (95% HPD, September 2006 to May 2007), which was only slightly later than the clinically-estimated root date (S1 Table). Proviruses persisting on ART interspersed throughout the whole tree, with the oldest dating to March 2007 (Fig. 9B). We observed a number of long-lived clones, including one that was recovered at every on-ART time point (Fig. 9B; black arrow). Proviral age distributions remained consistent over time on ART (Kruskal-Wallis p = 0.5; Fig. 9D), with no evidence of changing population structure (AMOVA p = 0.7; CC mean p = 0.7; Fig. 9E). Only one recombinant proviral sequence was isolated from participant 6, whose parents dated to the two years prior to ART (S4B Fig).

#### Sensitivity analyses and alternative approaches: phylogenetic inference using gag

Though *env-gp120* is commonly used for within-host evolutionary studies [6, 9, 10, 40], evolution in this region is characterized by (sometimes dramatic) genetic bottlenecks largely imposed by the evolving antibody response [41]. To confirm that our choice of HIV region did not unduly influence our results, we additionally dated participant 1, 3 and 7’s datasets using *gag* (as pre-ART *gag* sequences had previously been collected for these individuals [32]).

We begin with participant 7, who was not included in the primary analysis as proviruses were only sampled at a single on-ART time point, but whose data is nevertheless relevant for this validation, and provides additional insight into on-ART proviral genetic composition (Table 1; S5 Fig). We inferred phylogenies relating newly-collected *env-gp120* and *gag* on-ART proviral sequences (amplified independently) to the existing pre-ART plasma HIV RNA sequences [32] (see S5B Fig and S5C Fig for example trees and divergence plots, and S1 table for summary statistics). The age distributions of distinct proviruses sampled on ART did not significantly differ based on which gene was used for dating (Mann-Whitney p = 0.07; S5D Fig). *Env-gp120 and gag* proviral clonal profiles were also highly comparable, even though these regions were amplified separately (S5E Fig). Of the five *gp 120* recombinant proviral sequences recovered during ART in participant 7, none contained parents that dated to substantively different eras of infection (S6 Fig). No recombinant *gag* sequences were identified.

*Gag* analysis also corroborated the original observations for participants 1 and 3. For participant 1, the percentage clonality based on *gag* was 23% (versus 33% for *env-gp120)* while for participant 3 it was 39% (versus 58% for *env-gp120),* where clones again increased over time, and were observed across multiple time points (S7 Fig). For participant 1, *gag* analysis also confirmed that plasma rebound HIV sequences were significantly younger than, and represented a distinctive population compared to, the proviral pool that persisted during ART (S8E Fig; both AMOVA and CC mean p = 0). *Gag* analysis also corroborated the independent reactivation of a distinct and slightly more ancestral proviral lineage at the second rebound sampling time point in April 2011 (S8B Fig, bracket). It also confirmed that persisting proviruses had integrated throughout the entire course of untreated infection, and that proviral age distributions were stable over time on ART (Kruskal-Wallis p = 0.6) with no evidence of temporal population structure (AMOVA p = 0.6, CC mean p = 0.62) (S8D Fig, S8E Fig). For participant 3, *gag* analysis similarly confirmed that proviral ages were stable during ART (Kruskal-Wallis p = 0.8) with no evidence of temporal population structure (AMOVA p = 0.74, CC mean p = 0.97) (S9E Fig).

Importantly, the integration date distributions of on-ART sequences that were inferred from *env-gp120* versus *gag* were highly comparable. The sole exception was participant 1’s initial rebounding population, where *env-gp120* analysis returned on average older integration dates than *gag* analysis (p = 0.005, S10A Fig). Other than this, *env-gp120 and gag* analysis produced similar proviral integration dates for all 7 proviral time points analyzed for participants 1 and 3, and the remaining two rebound time points analyzed for participant 1 (all p ≥ 0.1, S10A Fig, S10B Fig, S10C Fig). No recombinant *gag* sequences were identified in participants 1 and 3. Overall, these observations indicate that our findings are not majorly influenced by the HIV region studied.

### Evolution in HIV coreceptor usage as a non-phylogenetic validation

As there is uncertainty in phylogenetic reconstruction, we also corroborated our findings using a “tree-free” approach. To do this we leveraged the shifts in HIV coreceptor usage that occurred over time in participants 1, 2 and 4 (coreceptor shifts did not occur over the observation period in the other participants). We inferred coreceptor usage from the V3 region of *env-gp120* sequences using the geno2pheno [coreceptor] algorithm, which assigns each sequence a “False Positive Rate (FPR)” between 0 and 100, which represents the likelihood that a CCR5-using virus is misclassified as CXCR4-using (i.e., sequences with low FPR are more likely to be CXCR4-using). For participants 1 and 2, coreceptor usage shifted from X4 to R5 during untreated infection (participant 1 ‘s shift was previously documented [32]), while participant 4 had a minority X4 population in early infection, that steadily became more dominant (S11 Fig).

In all three participants, the coreceptor usage results corroborated the phylogenetic ones. In participant 1, FPR distributions differed significantly between rebound HIV and proviruses persisting longitudinally on ART (Mann-Whitney p = 0.002 for comparison of all rebound viruses to all proviruses; p = 0.003 for comparison of initial rebound viruses to all proviruses). In contrast, yet consistent with the tree-based analyses, proviral FPR distributions were stable over time in both participants 1 and 2 (Kruskal-Wallis p = 0.3 [S11A Fig] and p = 0.2 [S11B Fig], respectively), but shifted over time in participant 4 (Kruskal-Wallis p = 0.02, where the average FPR was significantly higher at the latest time point compared to one of the earlier ones; post-test p = 0.04 S11C Fig).

### Temporal stability of on-ART proviral diversity: cross-participant analysis

**Figure 10:**
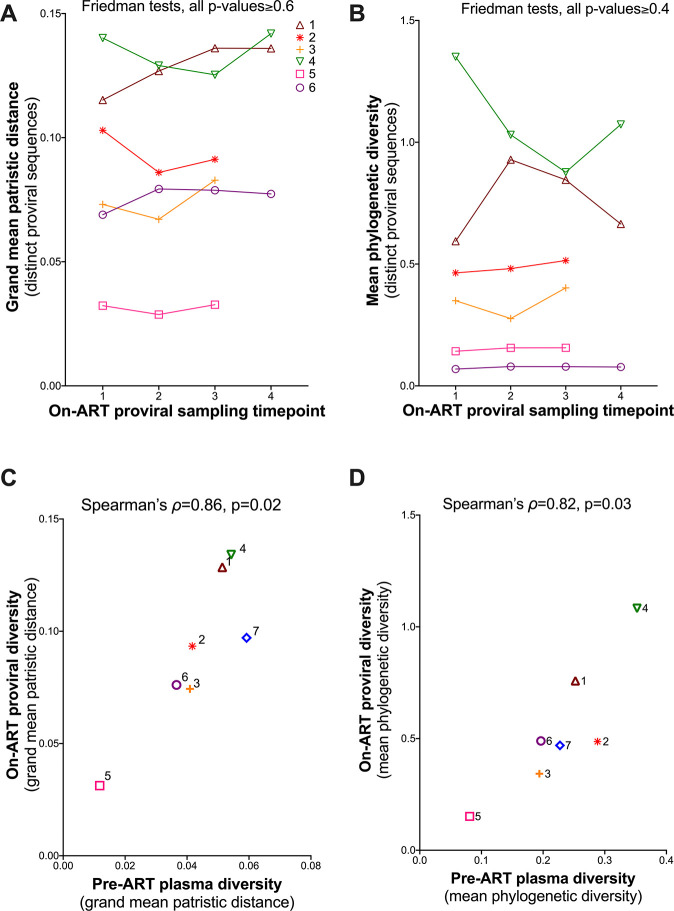
Proviral diversity and dynamics during ART. (A) Grand mean within-host patristic distance separating all pairs of distinct proviral sequences per time point, with a line linking each participant’s values. (B) same as A, but expressed in terms of participants’ mean phylogenetic diversity of distinct sequences. P-values were computed using a Friedman test applied to the first three time points for all participants, as well as to all four time points for participants 1,4 and 6. (C) Relationship between pre-ART plasma HIV RNA and on-ART proviral diversity, expressed in terms of grand mean patristic distance. (D) same as (C) but where diversity is expressed in terms of mean phylogenetic diversity.

As one of our major objectives was to investigate proviral genetic stability (in terms of distinct lineages) during the initial years of ART, we concluded by testing for trends in overall within-host proviral diversity in all participants over time. We used two metrics: the grand mean patristic distance (Fig. 10A), and the mean phylogenetic diversity (Fig. 10B), computed from all distinct, non-recombinant proviral sequences isolated per time point. For grand mean patristic distance, a Friedman test comparing the first three time points across all six participants yielded p = 0.6, while comparison across all four time points for participants 1,4 and 6 yielded p = 0.7 (Fig. 10A). Corresponding values for mean phylogenetic diversity were p = 0.4 and p = 0.7 (Fig. 10B). These observations further support the notion that proviral diversity is largely maintained during the initial years of ART.

Of note, the diversity of distinct proviral lineages persisting on ART correlated strongly with the overall plasma HIV diversity generated during untreated infection: when measured as grand mean patristic distance, Spearman’s correlation yielded ρ = 0.86, p = 0.02 (Fig. 10C); when measured as mean phylogenetic diversity, Spearman’s correlation yielded ρ = 0.82, p = 0.03 (Fig. 10D).

## Discussion

We reconstructed within-host HIV evolutionary histories from pre-ART plasma and on-ART proviral sequences sampled over a median of 14 (range 9–23) years in seven participants. These analyses can reveal the lineage origins – and ages – of proviruses persisting on ART, as well as insights into the temporal stability of the on-ART proviral pool in terms of its genetic diversity, composition and age distribution. In these analyses, clones were represented as a single sequence per time point so that the results were not influenced by clonal expansion. This was important because, in 4 of 6 participants, the proportion of clonally-expanded (defined as *env-gp-120*-identical) proviruses increased over time during ART (Fig. 2). Consistent with previous reports, clones persisted long-term (one clone was recovered at all time points over an 8-year period in participant 5), “waxed and waned” over time (e.g., participants 5 and 6), and dominated in some cases (participants 3 and 6’s proviral pools were > 50% clonal) [20, 21, 42–48].

Despite increasing clonality, the distinct proviral sequences comprising the proviral pool were highly stable in terms of genetic diversity, composition and age distribution. Though we found no broad evidence supporting the loss of proviral diversity over time, we did detect a modest yet statistically significant shift in proviral composition in participant 4, for whom the proviruses sampled 12 years post-ART were on average older, and exhibited significantly different population structure, than those sampled in the earlier years of ART. We do not believe that this is a sampling artifact (as proviruses were sampled twice in year 12 of ART, with consistent results). While we cannot absolutely exclude the possibility that “old” proviruses were newly seeded into the reservoir during ART due to ongoing reactivation of these lineages, the more plausible explanation is that “younger” proviruses (i.e., those seeded just prior to ART initiation) were preferentially eliminated during the initial years of ART. This gradually shifted the balance towards older, more long-lived proviruses, which were then more likely to be detected using the limiting-dilution approaches used here. This observation is consistent with a study of 4 individuals with HIV subtype C with longitudinal on-ART sampling, that also suggested that “younger” proviruses were preferentially eliminated during these initial years [6]. Of note, this participant had the longest follow-up of any individual in the study, which may have allowed the opportunity to observe this phenomenon.

Our study also indicates that the replication-competent HIV reservoir in blood (measured as HIV sequences that emerged in plasma post-ART in participants 1 and 5), represents a genetically restricted subset of the overall proviral pool, which is predominantly defective. Consistent with prior studies [5–7, 9–12], participants’ on-ART proviral pools ranged from modestly (e.g., participant 4) to substantially (e.g., participant 3) skewed towards viral variants that “dated” to the years immediately preceding ART, which is consistent with continual reservoir seeding – yet relatively rapid turnover – during untreated infection. By contrast, the plasma HIV sequences that emerged post-ART were a restricted subset that exclusively “dated” to the years immediately prior to ART. This suggests that replication-competent reservoir sequences older than this had either already been eliminated (or were extremely rare), or could not reactivate (e.g., due to integration into inaccessible chromatin [49]). Participant 1’s data also suggested that, during extended ART interruption, viral rebound occurs in sequential “waves” of reactivation from individual reservoir cells (or clonal populations), as evidenced by the new emergence of slightly more ancestral viral sequences 6 months into the treatment interruption. Participant 1 ‘s data are also consistent with studies from SIV that show that rebound viruses can re-seed the reservoir if rebound viral loads reach pre-ART levels [50], which occurred in this case.

Of note, recombinant proviruses were identified in nearly all participants, and recombinant HIV RNA sequences also emerged in plasma in participant 1 after ART interruption. All but two of these sequences represented mosaics of sequences that had plausibly circulated at the same time. Nevertheless, we did identify one provirus each from participants 2 and 4 whose parents dated to substantially different infection eras. This suggests that reservoir cells can become superinfected with HIV reactivated from an older era, an observation that to our knowledge has never been empirically observed, though mathematical modeling has suggested that this phenomenon does occur, and that recombination represents another method of survival of latent HIV genomes [39]. For participant 1, whose plasma HIV RNA rebound following ART interruption included recombinant sequences, no proviruses were identified that exactly matched a recombinant sequence (though note that proviral sampling occurred some years after the rebound). While this suggests *de novo* generation of recombinants during rebound, we cannot exclude the possibility that matching proviruses did exist in blood but were not detected at time of sampling, or that they had existed previously but were later eliminated, or that recombinant proviruses resided in tissue. The source of recombinant viruses during rebound events therefore remains an open question [31].

Our study has some caveats and limitations. All participants were women. Though there is no evidence that men and women differ in terms of rates of viral evolution [32], nor on-ART proviral genetic composition and age distribution [6], there is evidence that *ex vivo* reactivation potential and residual immune activation differ by sex [51–53]. Due to very limited sample availability (only 10 million PBMCs per proviral time point), we performed sub-genomic amplification, as near-full-genome HIV amplification would likely have generated many sequences with various large deletions in *env-gp120* (and/or *gag)* that could not be phylogenetically “dated”. We cannot therefore discriminate intact from defective proviruses. In fact, using data from another study [30], we estimate a 22% overall average likelihood (range 2–35% depending on the participant) that an intact *env-gp120* sequence comes from a genomically intact provirus. Because we only sequenced part of the HIV genome, we also cannot definitively characterize proviruses as clonal, which would require full-genome sequencing and integration site characterization. We also acknowledge that sequences isolated only once may still be part of a clonal set [54]. Because biological material was so limited, we could not perform reservoir size quantification, and since we isolated proviruses directly from PBMCs, the type of cell that harbored the isolated proviruses is unknown.

Despite these limitations, our study has some strengths and provides new insights into the within-host evolutionary origins and temporal stability of proviral lineages on ART, along with the origins of viruses emerging from the blood reservoir. We took various steps to validate our methods and estimate error, including conditioning our analyses across a distribution of trees to mitigate uncertainty in within-host phylogenetic reconstruction, validating our tree-based metrics using a second HIV region *{gag),* and confirming our results using a method that did not involve phylogenetic reconstruction (HIV coreceptor usage inference). Our study also boosts the representation of women living with HIV subtype B, who are under-represented in the within-host HIV evolutionary reservoir dynamics literature.

In conclusion, our results confirm that the diversity of proviruses persisting on ART, which are largely genetically defective [13–15], broadly reflects the extent of within-host HIV evolution prior to ART [6, 7]. We also observed that the clonal expansion that commonly occurs during the initial years of ART is not appreciably accompanied by the loss of distinct proviral lineages during this time. In fact, on-ART proviral genetic composition remained remarkably stable, with the exception of participant 4, in whom we found evidence of preferential elimination of “younger” proviruses (those that integrated near ART initiation) by the twelfth year of ART. Our analyses of recombinant sequences also enrich our understanding of HIV persistence by suggesting that reservoir cells can become superinfected with HIV reactivated from older infection eras, yielding mosaics of “older” and “younger” sequences. Finally, we observed that the replication-competent reservoir (studied here as post-ART rebound HIV sequences) comprise a genetically restricted, “younger” subset of all proviruses persisting in blood. This suggests that cure strategies will need to eliminate a reservoir whose key characteristics may differ from those of the overall proviral pool.

## Materials and methods

### Ethics statement

Institutional review boards at each WIHS clinical research center approved the protocol and all participants provided written informed consent. Additionally, this nested sub-study was approved by the institutional review boards at Providence Health Care/University of British Columbia, and Simon Fraser University (IRB# H19–01769).

### Study population

The Women’s Interagency HIV Study (WIHS) was a prospective, multi-center, cohort study of US women living with or without HIV [55–57]. WIHS participants were recruited at 10 sites over 4 time periods starting in 1994. While the WIHS study ended in 2019, most participants continue to be followed under similar protocols as part of the MACS/WIHS Combined Cohort Study (MWCCS) [58].

Eligibility criteria and study protocols for the WIHS have been previously described [55–57]. Briefly, data were collected using structured in-person interviews and standardized physical and laboratory assessments, with study visits occurring every six months. Eligible women had documentation of reactive anti-HIV serology and if positive, a confirmatory test or if they were HIV-seronegative had risk factor(s) for HIV exposure. Baseline sociodemographic characteristics and HIV risk factors were similar between HIV-seropositive and HIV-seronegative women.

We studied seven WIHS participants with the following criteria: documented HIV seroconversion during follow-up, initiated ART during chronic infection, at least 4 longitudinal pre-ART plasma samples available, and on-ART peripheral blood mononuclear cell (PBMC) samples available. These seven participants represented all WIHS participants who met these criteria. HIV infection dates were calculated as the midpoint between last negative and first seropositive WIHS study visits. At the time of the last proviral sampling, participants were a median 53 (range 49–54) years of age and had been receiving ART for a median of 9 years (range 2.8–12.3 years). In total, we collected longitudinal HIV RNA sequences over a median of 9 (range 2–13) study visits per participant spanning a median of 9 (range 0.8–11.8) years of untreated infection. Plasma HIV RNA sequences for participants 1,3 and 7 were published previously [32]. In addition, we collected single-genome-amplified proviral sequences over a median 3 (range 1 –4) time points per participant spanning a median 8.7 (range 2.8–12.3) years on-ART (only 10 million PBMCs per on-ART time point were available for analysis). We also isolated plasma HIV RNA sequences from participant 1 at three time points after ART was interrupted, and plasma HIV RNA sequences from participant 5 when viral control was initially being lost.

### HIV amplification, sequencing, and curation

Total nucleic acids were extracted from plasma using the NucliSENS EasyMag (BioMerieux, Marcy-l’Etoile, France), and DNase I-digested (New England Biolabs) if the plasma viral load (pVL) was < 2000 copies/mL, to minimize the risk of amplifying proviral DNA. Genomic DNA was extracted from 10 million peripheral blood mononuclear cells (PBMC) per time point using the QIAamp DNA Mini Kit (Qiagen). Single-genome amplification of a subgenomic HIV region (*env-gp120*, and *gag* where applicable) was performed as follows. For plasma HIV RNA, cDNA (generated using NxtScript reverse transcriptase; Roche) was generated using HIV-specific primers and endpoint-diluted such that subsequent nested PCR reactions (generated using the Expand High Fidelity PCR system; Roche) yielded no more than 30% positive amplicons [12, 46]. Proviral DNA extracts were similarly endpoint-diluted and amplified by nested PCR. For *env-gp120,* first round primers were: 5’-TTAGGCATCTCCTATGGCAGGAAGAAGCGG-3’ (forward; HIV reference strain HXB2 genomic nucleotide coordinates 5957–5986) and 5’-TAAGTCATTGGTCTTAAAGGTACC-3’ (reverse; HXB2 9038 – 9015); second round primers were 5’- GGCCGCGTCGACAAGAGCAGAAGACAGTGGCAATGA-3’ (forward; HXB2 6194–6228) and 5’- GGCCGCGGATCCGTGCTTCCTGCTGCTCCCAAGAAC-3’ (reverse; HXB2 7823 – 7787) [32], For *gag,* first round primers were 5’- AAATCTCTAGCAGTGGCGCCCGAACAG-3’ (forward; HXB2 629–649) and 5’-TAACCCTGCGGGATGTGGTATTCC-3’ (reverse; HXB2 2849 – 2826); second round primers were 5’-GCAGGACTCGGCTTGCTGAA-3’ (forward; HXB2 691 –710) and 5’-TATCATCTGCTCCTGTATC-3’ (reverse; HXB2 2343 – 2325). Negative controls were included in every run. For extracts from plasma samples with low pVL, we confirmed that HIV RNA amplification did not occur in the absence of reverse transcription. Amplicons were sequenced on a 3730×1 automated DNA sequencer using BigDye (v3.1) chemistry (Applied Biosystems). Chromatograms were analyzed using Sequencher (v5.0/v5.4.6) (GeneCodes). Sequences with with nucleotide mixtures were excluded from analysis.

Hypermutated sequences were identified using Hypermut [59]. Sequences exhibiting evidence of putative within-host recombination were identified using RDP4 v4.1 [38]. This program identifies the putative major and minor parent sequences for each recombinant, along with their approximate breakpoints, allowing us to “date” each sequence fragment by assigning the date of origin of its parent. Hypermutant and recombinant sequences, along with those with minor defects (e.g., small deletions) were retained in the analysis of clonality, but excluded from phylogenetic inference. Sequence alignments were performed in a codon-aware manner using MAFFT v7.471 [60]. Alignments were inspected and manually edited in AliView v1.26 [61]. Following automated model selection using ModelFinder [62], between-host phylogenies were inferred by maximum likelihood methods using IQ-TREE2, with the ultrafast bootstrap option (1,000 bootstraps) [63, 64].

### Within-host phylogenetic inference and proviral dating

Within-host phylogenies were inferred from *env-gp120 and* gag sequence alignments comprising all plasma and proviral sequences collected per participant. To mitigate uncertainty in phylogenetic reconstruction, we inferred a median 4,500 (range 1,500 – 15,000) phylogenies per participant using Bayesian approaches, and conditioned results across all trees. To do this, we first reduced each within-host nucleotide sequence alignment to distinct, intact, non-recombinant sequences and determined the best-fitting nucleotide substitution model for each alignment using jModelTest v2.1.10 (S1 Table) [65]. Next, Markov chain Monte Carlo (MCMC) methods were used to build a distribution of phylogenies per participant without enforcing a molecular clock. Two parallel runs with MCMC chains of a median 30 million generations, sampled every 10,000 generations, were performed in MrBayes, v3.2.5 [66] using the best-fitting substitution model and model-specific or default priors. Convergence was assessed by ensuring the standard deviation of split frequencies was < 0.04, the effective sampling size of all parameters was the ≥ 200, and by visual inspection of parameter traces using Tracer v1.7.2 [67]. In the single case where convergence was not achieved (participant 1, *env-gp120*), the run was terminated at 100 million generations. The first 25% of MCMC iterations were discarded as burn-in, yielding a minimum of 1,500 (maximum 15,000) *env-gp120 and gag* phylogenies per participant (S1 Table).

We then inferred the integration dates of on-ART sequences of interest using a phylogenetic approach [7]. First, each tree was rooted at the location that maximized the correlation between the root-to-tip distances of the pre-ART plasma HIV RNA sequences and their sampling dates, which represents the most recent common ancestor of the dataset. We then fit a linear model relating the root-to-tip distances of pre-ART plasma HIV sequences to their collection dates, where the slope of this line represents the average within-host gene-specific evolutionary rate, and the x-intercept represents the root date. Model fit was assessed by comparing its Akaike Information Criterion (AIC) to that of the null model (a zero slope). In order to pass quality control (QC), a phylogeny required a ΔAIC ≥ 10 and an inferred root date that was prior to the first plasma sampling. A median 3,842 (range 1,278–7,218) phylogenies per participant passed QC (S1 Table). The linear models from these QC-passed phylogenies were used to convert the root-to-tip distances of on-ART sequences of interest to their integration dates. The custom R script for this method is available at https://github.com/cfe-lab/phylodating. The script was implemented via GNU parallel [68] to run more than one tree at a time. The integration dates were then averaged across all QC-passed phylogenies per participant to produce mean integration date estimates and 95% highest posterior density (HPD) estimates, computed using R package HDInterval (version 0.2.2).

As phylogenies were inferred using distinct within-host HIV sequences only, identical sequences were then grafted back onto the phylogenies using the add.tips function in the R package phangorn, v2.8.1, as appropriate for each analysis [69]. The example phylogeny shown for each participant was the highest likelihood tree among those that passed QC. Phylogenies and highlighter plots were plotted using the R (v4.1.2) package ggtree version 3.21. Node support values were derived from Bayesian posterior probabilities generated from the consensus trees.

### Proviral population structure and diversity analyses

Within-host proviral populations sampled during ART were tested for evidence of population structure using Analysis of Molecular Variance (AMOVA) [70], which is a genetic-distance based test, and the Correlation Coefficient (CC) test [71], which is a tree-based test. These were chosen because they can test for population structure across more than two time points. Tests were performed on distinct sequences per on-ART time point. AMOVA was implemented in the R package pegas, v1.1 [72] using the K80 substitution model [73], where statistical significance was assessed via 1,000 permutation tests. CC test statistics were averaged over all QC-passed phylogenies per participant, using a custom R script available at https://github.com/brj1/HIVCompartmentalization. A dataset was classified as having evidence of population structure if both tests returned p < 0.05 (where, for CC, this was defined as a mean p < 0.05 overall QC-passed trees).

Within-host proviral diversity was quantified using two metrics: the grand mean patristic (tip-to-tip phylogenetic) distance, calculated as the mean patristic distance between all pairs of distinct sequences per time point averaged over all QC-passed phylogenies, and the mean phylogenetic diversity, calculated by summing the edge lengths of all distinct sequences per time point, averaged over all QC-passed phylogenies [74]. Both metrics were computed using custom R scripts available at https://github.com/brj1/HIVCompartmentalization. All tests of significance were two-tailed (except compartmentalization tests which are one-tailed), with p < 0.05 denoting statistical significance. All other statistical analyses were performed in Prism, v9.0 (GraphPad Software).

### HIV coreceptor usage

Coreceptor usage was predicted from the V3 region of HIV-1 *env-gp120* sequences using geno2pheno [coreceptor] (https://coreceptor.geno2pheno.org) [75]. This support vector machine-based approach assigns each V3 sequence a false-positive rate (FPR) that represents the probability of falsely classifying an R5 virus as X4. We considered V3 sequences with FPR ≤ 10% as X4 and those with FPR ≥ 10% as R5.

## Figures and Tables

**Table 1:** Participant information, HIV sampling and sequencing details

ID	Estimated date of infection	Duration of uncontrolled infection (years)	No. of pre-ART plasma HIV RNA time points	pre-ART plasma HIV *env-gp120* sequences Total N (distinct N; %)	ART initiation date	No. of post-ART plasma HIV RNA time points^^^	Post-ART plasma HIV RNA *env-gp120* sequences Total N (distinct N; %)	Years of ART until last proviral sampling	No. of on-ART proviral time points	On-ART *env-gp120* proviral sequences Total N (distinct N; %)
1	Dec 1995	12	13	207 (207; 100%)	Jan 2008	3	114 (97; 85%)	10.3	4	171 (114; 67%)
2	Jan 2003	9	10	239 (227; 95%)	Jan 2012	-	-	6.8	3	95 (79; 83%)
3	Jul 2002	5.5	9	140 (132; 94%)	Jan 2008	-	-	8.8	3	150 (63; 42%)
4*	Jul 1995	10.9	9	195 (182; 93%)	Jun 2006	-	-	12.3	4	182 (165; 91%)
5	Mar 2008	1.9	2	50 (45; 90%)	Feb 2010	1	4 (2; 50%)	8.7	3	110 (71; 65%)
6	Aug 2006	3.9	6	80 (73; 91%)	Jul 2010	-	-	8.3	4	176 (84; 48%)
7	Sep 1999	11.9	11	181 (181; 100%)	Aug 2011	-	-	2.8	1	42 (25; 60%)

* Clinical records indicated that participant 4 initiated ART in 2003, but no reductions in plasma viral load (pVL) were observed until June 2006. For this reason, we considered June 2006 as this participant’s effective ART start date. Also see Fig. 7A.

^ Post-ART plasma HIV RNA sampling time points include a prolonged plasma rebound event (participant 1) and initial loss of viral control (participant 5), as shown in Fig. 3A and Fig. 8A, respectively.
